# Single sessions of transcranial direct current stimulation and transcranial random noise stimulation exert no effect on sleepiness in patients with narcolepsy and idiopathic hypersomnia

**DOI:** 10.3389/fpsyt.2023.1288976

**Published:** 2023-12-11

**Authors:** Michaela Hohenester, Berthold Langguth, Thomas Christian Wetter, Peter Geisler, Martin Schecklmann, Andreas Reissmann

**Affiliations:** ^1^Department of Psychiatry and Psychotherapy, University of Regensburg, Regensburg, Germany; ^2^Department of Hematology and Oncology, Krankenhaus der Barmherzigen Brüder Regensburg, Regensburg, Germany

**Keywords:** anodal tDCS, tRNS, hypersomnia, narcolepsy, idiopathic hypersomnia, vigilance, sleepiness

## Abstract

**Background:**

Hypersomnia poses major challenges to treatment providers given the limitations of available treatment options. In this context, the application of non-invasive brain stimulation techniques such as transcranial electrical stimulation (tES) may open up new avenues to effective treatment. Preliminary evidence suggests both acute and longer-lasting positive effects of transcranial direct current stimulation (tDCS) on vigilance and sleepiness in hypersomniac patients. Based on these findings, the present study sought to investigate short-term effects of single sessions of tDCS and transcranial random noise stimulation (tRNS) on sleepiness in persons suffering from hypersomnia.

**Methods:**

A sample of 29 patients suffering from narcolepsy or idiopathic hypersomnia (IH) was recruited from the Regensburg Sleep Disorder Center and underwent single sessions of tES (anodal tDCS, tRNS, sham) over the left and right dorsolateral prefrontal cortex on three consecutive days in a double-blind, sham-controlled, pseudorandomized crossover trial. The primary study endpoint was the mean reaction time measured by the Psychomotor Vigilance Task (PVT) before and directly after the daily tES sessions. Secondary endpoints were additional PVT outcome metrics as well as subjective outcome parameters (e.g., Karolinska Sleepiness Scale; KSS).

**Results:**

There were no significant treatment effects neither on objective (i.e., PVT) nor on subjective indicators of sleepiness.

**Conclusion:**

We could not demonstrate any clinically relevant effects of single sessions of tDCS or tRNS on objective or subjective measures of sleepiness in patients with hypersomnia. However, we cannot exclude that repeated sessions of tES may affect vigilance or sleepiness in hypersomniac patients.

## 1 Introduction

Narcolepsy and idiopathic hypersomnia (IH) are yet incurable neurological disorders characterized by excessive daytime sleepiness (EDS) and sleep attacks despite quantitatively sufficient sleep at night for a period of at least 3 months (1). Sudden involuntary sleep attacks may occur repeatedly even under inadequate or potentially dangerous circumstances. The prevalence of narcolepsy (both type 1 and 2) is estimated at 0.03–0.06% (2, 3), whereas IH is even more rare (4, 5). In addition to EDS, the classic symptom of narcolepsy type 1 (NT 1) is cataplexy. Cataplexy is defined as a sudden loss of muscle tone of differing severity occurring in striated muscles except for the respiratory muscles. Cataplexy is provoked by strong emotions and has a short duration of usually less than 2 min (1, 6). Additional symptoms of narcolepsy (both types) include the potential occurrence of sleep paralysis, defined as the transient inability to move during the onset of sleep, and hypnagogic hallucinations, characterized by commonly frightening dream-like visual, acoustical or sensory misperceptions (7). Additionally, disrupted nocturnal sleep patterns and a decline in sleep quality, which tend to worsen with the progression of the disease, can further burden individuals afflicted by these conditions. A heterogeneous pattern of clinical symptoms and the absence of cataplexy are typical for NT 2 and IH (4, 8). NT 1 is assumed to result from a reduction or absence of the hypothalamic neuromodulatory peptide hypocretin-1 that controls and modifies the sleep–wake cycle (9–12), whereas the etiology of NT 2 and IH is still largely unknown (12, 13). An irregularity in rapid eye movement (REM) sleep, characterized by a shift in the timing of REM sleep characteristics within the sleep cycle or during waking periods, is a typical symptom of narcolepsy, but not of IH (8, 14–16). All three diagnostic groups (NT 1, NT 2, and IH) share the characteristic finding of a short mean sleep latency of less than 8 min in the multiple sleep latency test (MSLT). The occurrence of 2 or more sleep onset REM sleep periods (SOREM) with a latency of less than 15 min from sleep onset in the MSLT discriminates narcolepsy (both NT 1 and NT 2) from IH (1, 17–19).

At the brain level, mounting neuroimaging evidence has suggested several brain regions to play a role in the pathophysiology of EDS disorders (20, 21). Hence, structural and functional findings imply that the frontal cortex plays a crucial role in narcolepsy. Studies have demonstrated frontal cortex hypoactivity to be associated with the pathophysiology of narcolepsy. This hypoactivity also affects several diencephalic and cortical areas connected to the so-called orexin network. These findings underscore the potential importance of the frontal cortex in regulating sleep–wake states and emotional processes that may play a role in the manifestation of narcolepsy symptoms. Some of the neuroimaging studies on IH suggest a dysfunction or alterations in the frontal cortex. Reduced regional cerebral blood flow (rCBF) in the medial prefrontal cortex and posterior cingulate cortex was observed in a SPECT study (22). Taken together, there is preliminary evidence to suggest that hypofrontality plays a role in both narcolepsy and IH.

Pharmacological treatment options of EDS include amphetamine- or other-type stimulants (23, 24). However, patients with narcolepsy and IH frequently experience loss of effectiveness of stimulant drugs, drug intolerance and dose limiting side-effects (24–27). Due to these limitations, there is an urgent need for further treatment options for patients with hypersomnia, and transcranial electrical stimulation (tES) has been suggested as a potential new non-pharmacological therapy (28–31). tES represents an emerging non-invasive neuromodulatory technique enabling electrical stimulation of the brain (32). Amongst others, transcranial direct current stimulation (tDCS) and transcranial random noise stimulation (tRNS) fall into this category. In both procedures, weak electric currents with intensities of 1–2 mA are applied to the human brain by pairs of conductive electrodes (32, 33). Anodal tDCS typically increases neuronal excitability in the area under the electrode, while cathodal tDCS typically reduces excitability (32, 34–38). While tDCS operates with continuous current, tRNS is an oscillatory stimulation technique that affects electrical brain activity by using randomly generated noise current (39). Importantly, tRNS is polarity-independent and has been shown to increase neuronal excitability under both electrodes when applied in the high frequency spectrum between 100 and 640 Hz (39, 40), hence mimicking the effect of anodal tDCS. Both tDCS and tRNS have been shown to produce short- or long-lasting after-effects on neuronal excitability depending on a variety of factors such as the placement and polarity of stimulation electrodes (34, 41), the employed current density (41, 42) or frequency spectrum (43). Likewise, the duration of the stimulation sessions (44, 45), the repetition of the treatment (38, 46) and intraindividual characteristics (47, 48) were shown to be of importance in determining stimulation effects. In conclusion, tDCS and tRNS can induce and contribute to neuromodulatory processes similar to long-term potentiation or long-term depression (40, 43, 49, 50). Due to its neuromodulatory effects, tDCS represents a promising non-pharmaceutical treatment approach in the context of several neuropsychiatric disorders and has a growing evidence base (51). tRNS, when compared to tDCS, has been shown to lead to larger neuromodulatory effects in terms of motor cortex excitability (52, 53). Moreover, there is comparative evidence to suggest tRNS may also lead to larger neuromodulatory effects when studied in the context of clinical applications in neuropsychiatric settings. As an example, a comparative study of tRNS and tDCS in Tinnitus patients showed significant suppressive effects of tRNS on Tinnitus loudness, while tDCS was without effect (54). When safety standards of tES are observed, tDCS and tRNS are free of serious adverse effects, pain and long-term impairment. As a minor side effect subjects could feel sensations such as itching and tingling limited to the duration of the stimulation (39, 43, 55, 56). Sham stimulation can be reliably realized by using weak electrical currents only at the beginning and at the end of tES (32, 43, 57). Sham stimulation causes mild sensory feelings under the electrical stimulus electrodes, but does not induce any detectable neuromodulatory effects (58, 59).

The use of tES in the modulation of sleep and wakefulness relies on the targeted induction of local changes in cortical excitability, potentially leading to improvements in sleep patterns. The application of tES has been linked to the modulation of specific brain oscillations, such as slow wave activity during non-REM sleep (60). This modulation is thought to occur through a “top-down” pathway involving the frontal cortex and thalamus, emphasizing the role of fronto-thalamic feedback in sleep regulation (60, 61). While further research is required to fully understand its therapeutic potential, tES has demonstrated relatively minor side effects and holds promise for the treatment of sleep-related neuropsychiatric conditions (60).

Modulatory effects of tES on sleep and wakefulness in healthy adults, e.g., in terms of sleep duration, sleep efficiency and vigilance could be demonstrated in recent studies (29, 60, 62–64). Cheng et al. (63) were able to show that a single session of tDCS, applied over the left dorsolateral prefrontal cortex (DLPFC), led to improvements in several cognitive functions and subjective levels of wakefulness in sleep-deprived subjects for at least 2 h. Likewise, a recent review by Annarumma et al. (65) showed that tES has the potential to acutely enhance vigilance levels and reduce sleepiness: for example, acute effects of direct current include increased levels of vigilance, as reflected in improved stimulus detection during prolonged attentional tasks (66). Moreover, anodal tDCS has been shown to induce an EEG pattern of cortical arousal, potentially contributing to increased vigilance levels in healthy subjects. In a study of healthy subjects during a 30-h period of wakefulness by McIntire et al. (31), it was demonstrated that a single 30-min session of anodal tDCS over the PFC exhibited superior subjective and objective effectiveness in preventing sleepiness-related neurocognitive performance decrements compared to placebo stimulation and was comparable to caffeine (dose: 200 mg).

While there is convincing evidence regarding wakefulness-enhancing effects of tES in healthy subjects, there only is a paucity of corresponding research of tES effects in EDS disorders. Frase et al. (28) employed a six-day period of treatment, during which one patient with organic hypersomnia underwent three sessions of anodal tDCS, alternating with three sessions of sham stimulation on six consecutive days in phase I of the study. The stimulation parameters were identical as compared to the present study with the stimulation electrodes placed bi-frontally on the scalp (FP1/FP2 according to the international EEG 10-20-System) and the reference electrodes placed in a parietal position (P3/P4). Across the three tDCS treatment days, there were acute positive effects of tDCS on PVT reaction times as the variance of response speed (SD iRT) significantly improved after tDCS and deteriorated after sham stimulation. In phase II of the study of Frase et al. (28), two courses of three sessions of tDCS followed by a month of self-observation improved subjective vigilance (visual analog scale) and reduced the self-reported duration of daytime sleep. In contrast, the stimulation protocol used by Galbiati et al. (30) included repeated anodal tDCS over the left dorsolateral prefrontal cortex in eight patients with untreated IH (anodal stimulation electrode: left F3; 2 mA over the stimulation electrode; reference electrode: over the right orbit; 20 min per day; 3 stimulations per week for 4 weeks). Operationalized by the reaction time in the modified Attention Network Test, there was a significant improvement of attention functions and decrease in ESS scores in a pre−/post-comparison after the treatment series, suggesting beneficial therapeutic effects on sleepiness and performance in patients with untreated IH.

The present sham-controlled study examined short-term effects of tDCS and tRNS on sleepiness and sustained attention in patients with narcolepsy and IH. We chose to include tRNS as an additional stimulation mode, since evidence suggests that tRNS may be more effective in enhancing neuronal excitability (52, 53) and up to now no study made use of tRNS in hypersomniac patients. Given the limited evidence base in this field of study and uncertainties regarding the number of tES sessions required to induce sleepiness-modulating effects in hypersomniac patients, we investigated the exploratory hypothesis that single sessions of tES (with tDCS or tRNS) would lead to acute changes in both sustained attention and subjective levels of momentary sleepiness in hypersomniac patients.

## 2 Materials and methods

### 2.1 Sample

For this double-blind, sham-controlled and pseudo-randomized crossover trial, we recruited 29 patients with hypersomnia and excessive daytime sleepiness (EDS) from the Regensburg Sleep Disorder Center. The sample includes 27 patients with narcolepsy (14 NT 1 and 13 NT 2) and two patients with IH. The following *inclusion criteria* were applied:

- age between 18 and 75 years
- male or female gender
- established diagnosis of narcolepsy or IH based on International Classification of Sleep Disorders (Third Edition) criteria (1)
- stable drug or non-drug therapy of EDS and other narcolepsy symptoms for at least 4 weeks
- subjective burden of EDS as defined by an Epworth Sleepiness Scale [ESS; (67, 68); RRID:SCR_024211] score of at least 10. As a notable exception, one patient with a definite diagnosis of NT 2 and an ESS score below 10 was also included, as she suffered from comorbid conditions preventing her from falling asleep during daytime (i.e., Periodic Limb Movement Disorder, Chronic Pain Syndrome).

Patients were not included who fulfilled the following *exclusion criteria*:

- any exclusion criteria for tES (32)
- current substance abuse
- untreated and severe internal, neurological or psychiatric comorbidities.

The present study received approval from the local ethics committee in Regensburg (reference: 15-101-0295). After adequate explication, patients gave their written informed consent before participation in the study procedures.

### 2.2 Randomization and procedures

The study was conducted at the Regensburg Center of Neuromodulation, where patient recruitment took place between March and October 2016. Enrolled study patients underwent sessions of tES on three consecutive days in a double-blind, sham-controlled and pseudo-randomized crossover trial. Before participation in the trial, patients were informed about the presence of one sham stimulation in the course of the study. The randomization list, which specified the order of the different treatment conditions for each subject, was only known to the operators applying the actual tES treatments and who were not involved in rating and psychometric testing procedures. Unblinding took place after the completion of data entry in the employed statistical software. This procedure ensured the blinding of patients and examiners during the execution of study procedures.

Following the procedures outlined in Frase et al. (28), daily tES sessions were comprised of two stimulation periods of 13 min duration, with an intersession interval of 20 min (total tES session duration per day with preparation of electrodes: about 50 min). The first stimulation period started at noon between 11.30 am and 12.30 pm. During the tES stimulations, patients were positioned comfortably in a treatment chair in a semi-reclined position. As it could not be ensured to reliably prevent the patients from falling asleep during tES sessions, the study participants were allowed to spend the treatment time while being awake or asleep. Based on a self-report, it was documented whether the patients spent the treatment time awake or asleep. During treatment breaks, patients were instructed not to leave the building, to remain awake and to avoid caffeine, nicotine or other-type stimulants.

Stimulation modes were delivered by a battery-driven, micro-processor-controlled and CE-certified constant current stimulator (DC-Stimulator PLUS, neuroConn GmbH, Illmenau, Germany; RRID:SCR_015520). Electrode setup comprised two frontal sponge electrodes (5 × 7 cm, 10–20 electrode positions FP1 and FP2) and two parietal sponge electrodes (10 × 10 cm, P3 and P4) moistened with NaCl solution. While the frontal electrodes used the standard size for effective stimulation, parietal electrodes were increased in size in order to reduce current density to a level shown to be functionally inert to the cerebral cortex (69). Bi-frontal stimulation was selected to target the proposed ‘top-down’ pathway of sleep–wake regulation (60) and to keep the present procedures in line with those presented by Frase et al. (28). For tDCS, a current of 1 mA was applied over each anodal electrode (2 mA stimulator output, Y-cable split for stimulation and reference electrodes). For tRNS, the same setup with 100–640 Hz was used. For sham stimulation, we used the tDCS stimulation with 30 s fade-in and 30 s fade-out phase without active stimulation in between.

Directly before and after the daily tES sessions, psychometric ratings (Karolinska Sleepiness Scale, see below) and the Psychomotor Vigilance Task (PVT) developed by Dinges & Powell (70) were carried out. The test required subjects to monitor a display and to respond as fast as possible after the occurrence of a visual target. A single test session took 10 min. The test is known for its sound psychometric properties (70, 71) providing measures of both speed (inverse of the mean reaction time, mean iRT; standard deviation of the inverse reaction time, SD of iRT) and accuracy (number of lapses; number of errors). A lapse was defined as a reaction time longer than 500 milliseconds, and an error was defined as each reaction without a preceding visual stimulus or a reaction time shorter than 100 ms that exceeds the human performance limitations (72). In the present study, the portable version of the PVT (PVTL192, Ambulatory Monitoring Inc., Ardsley, NY; RRID:SCR_024213) with a test duration of 10 min and an interstimulus interval of 2–10 s was used. Additionally, subjects rated their momentary level of sleepiness with the Karolinska Sleepiness Scale before and after stimulation [KSS; (73, 74); RRID:SCR_024212]. Following the nights after each of the tES sessions, subjects were asked for the quality of their nighttime sleep using a 7-point Likert scale [ranging from 1 – “*Unaffected (very restful)*” to 7 – “*Severly impaired (not restful at all)*”].

While subjective ratings were obtained from all 29 patients, only 24 patients provided complete data of the PVT. This was due to logistical problems in the procedures, as PVT testing was conducted at the Regensburg Sleep Disorder Center (2 min walking distance from the neuromodulation unit) and some of the patients missed single assessments (hence dropping out of the data analysis). Statistical analysis of subjective ratings with the whole sample versus the subsample applying full PVT data showed no differences in results (data not shown), hence the results of the whole sample data will be presented.

Finally, during the last visit, patients were asked to guess on which day the sham treatment was applied. The sham stimulation was correctly identified by 44.8% of the patients, while 17.2% mistook the tDCS session and 37.9 the tRNS as the sham treatment.

### 2.3 Statistical analysis

Statistical analyses were performed using IBM SPSS Statistics for Windows [Version 28; (75); RRID:SCR_016479]. Due to the exploratory nature of the hypotheses, all inferential tests were conducted with a two-sided approach, and the significance threshold (α) was set at 0.05 to control the Type I error rate.

The primary endpoint was the PVT mean reaction time (mean iRT) *after* each tES session, as compared to the PVT mean reaction time assessed *before* the session. Secondary endpoints were the remaining PVT outcome metrics (standard deviation of the iRT, number of lapses, number of errors) *after* each tES session, as compared to the assessment *before* the session.

Additionally, ratings of momentary sleepiness assessed by the KSS *after* each tES were analyzed, as compared to KSS ratings *before* the session. Lastly, ratings of sleep quality for the night *after* the tES session were compared.

PVT and KSS outcome metrics were analyzed in a pre/post comparison on each day of study treatment (tDCS, tRNS, sham). For this purpose, a 3 × 2 two-factorial repeated measures analysis of variance (RM-ANOVA) was implemented to detect the effects of active treatment (tDCS, tRNS) and sham stimulation in a pre/post treatment design. For the ratings of sleep quality following each of the three tES sessions, a one-factorial RM-ANOVA was conducted. In case of violations of sphericity, as assessed with Mauchly’s Test of Sphericity, a Greenhouse–Geisser correction was performed (76). Likert-type items (KSS, item on sleep quality) were analyzed using parametric statistical procedures, as ANOVA procedures have been shown to be robust against violations of their assumptions (e.g., normality) even with single Likert-type items (77, 78). Additionally, effect size measures were calculated for pre/post treatment comparisons of PVT and KSS measures for all treatment conditions. The employed effect size measure was a measure of Cohen’s *d* paying attention to the repeated measures design, the correlation between measurements as well as the (pooled) standard deviations of the measurements [*d_RM, pooled_*; see (79), formulas 8 and 9]. *Post hoc* power analysis was conducted using the software MorePower 6.0 introduced by Campbell and Thompson [(80); RRID:SCR_024210].

## 3 Results

### 3.1 Patient characteristics

A total of 29 patients with a mean age of 42.86 ± 14.39 years and a gender ratio of 18:11 (female:male) were enrolled in the study (see also Table 1). A total of 27 patients (93.1%) showed clinical EDS values (ESS scores >10) indicating clinical levels of excessive daytime sleepiness, even though the majority of study patients (75.9%) received pharmacological treatment such as amphetamine- or other-type stimulants (e.g., modafinil, pitolisant, methylphenidate and sodium oxybate; see also Table 1). At the subgroup level, 84.6% of the patients diagnosed with NT 1, 64.3% of the patients with NT 2 and both patients suffering from IH were treated with medication.

**Table 1:** Patient characteristics.

Patient characteristics	Total (*n* = 29)	NT 1 (*n* = 14)	NT 2 (*n* = 13)	IH (*n* = 2)
Age (years), *M* ± *SD*	42.86 ± 14.39	40.14 ± 13.04	44.69 ± 16.64	50.00 ± 4.24
Sex (male), *n* (%)	11 (37.9)	5 (35.7)	5 (38.5)	1 (50)
Hypersomnolence				
Duration of symptoms (years), *M* ± *SD*	19.71 ± 14.52	20.82 ± 10.90	18.85 ± 18.67	17.50 ± 12.02
Duration of diagnosis (years), *M* ± *SD*	8.13 ± 7.84	11.19 ± 9.70	5.39 ± 4.38	4.50 ± 3.54
Latency of diagnosis (years), *M* ± *SD*	11.58 ± 12.86	9.64 ± 9.96	13.45 ± 15.81	13.00 ± 15.56
Symptoms				
Cataplexy, *n* (%)	14 (48.3)	14 (100)	–	–
Sleep paralysis, *n* (%)	20 (69.0)	13 (92.9)	7 (53.8)	0 (0)
Hypnagogic hallucinations, *n* (%)	22 (75.6)	11 (78.6)	10 (76.9)	1 (50)
Medication				
Stimulants, *n* (%)	22 (75.6)	9 (64.3)	11 (84.6)	2 (100)
Anticataplectics / Antidepressants, *n* (%)	7 (24.1)	5 (35.7)	2 (15.4)	–
Sedatives / hypnotics, *n* (%)	2 (6.9)	1 (7.1)	1 (7.7)	–

Patient characteristics of the overall patient collective (Total) and the diagnostic subgroups of narcolepsy type 1 (NT 1), narcolepsy type 2 (NT 2) and IH are expressed as n (%), or mean ± standard deviation.

### 3.2 PVT outcome measures

Concerning mean RT, descriptive analyses revealed minimal increases or decreases (<10 ms) in RT after the different stimulation conditions (see Table 2). The two-factorial RM-ANOVA showed no significant interaction between stimulation mode (tDCS, tRNS, sham) and time of measurement (pre/post stimulation) on mean RT values (Table 3). Likewise, neither the main effect for the stimulation mode nor that for time of measurement was significant (see Table 3). For none of the secondary PVT outcome metrics were there any significant main or interaction effects, as revealed through the conducted two-factorial RM-ANOVAs (see Table 3). In descriptive terms, there was a trend toward a higher number of errors after the tES stimulation sessions (see Table 2), as underlined by a near-significant main effect of Time of measurement in the corresponding RM-ANOVA model (*p* = 0.07, $\eta_{p}^{2}$ = 139; see Table 3).

**Table 2:** PVT reaction parameters before and after tDCS, tRNS and sham stimulation (*M* ± *SD*; [Cohen’s *d*_RM, pooled_]; *n* = 24).

PVT parameter	tDCS		tRNS		Sham	
	Pre	Post	Pre	Post	Pre	Post
Mean iRT (1/RT)	3.59 ± 0.67	3.62 ± 0.61	3.74 ± 0.54	3.67 ± 0.64	3.67 ± 0.59	3.74 ± 0.61
	[−0.058]		[0.112]		[−0.112]	
SD iRT	0.65 ± 0.14	0.65 ± 0.13	0.67 ± 0.14	0.61 ± 0.08	0.67 ± 0.14	0.64 ± 0.14
	[−0.042]		[0.425]		[0.249]	
Lapses	4.67 ± 8.40	3.50 ± 6.49	1.88 ± 2.94	3.08 ± 6.66	3.17 ± 5.09	3.42 ± 7.70
	[0.156]		[−0.193]		[−0.037]	
Errors	1.08 ± 1.02	2.37 ± 2.32	1.46 ± 1.72	2.83 ± 5.19	1.63 ± 1.69	2.04 ± 2.40
	[−0.684]		[−0.284]		[−0.198]	

**Table 3:** Results of two-factorial RM-ANOVAs with PVT outcome metrics.

	Factor	Test statistics				
		*df1, df2*	*F*	*p*	$\eta_{p}^{2}$	1-β
Mean RT	Stimulation mode	2, 46	2.720	0.08	0.106	0.512
	Time of measurement	1, 23	0.043	0.84	0.002	0.056
	Stimulation mode* Time of measurement	1.51, 34.82	0.722	0.46	0.030	0.165
SD RT	Stimulation mode	2, 46	0.414	0.66	0.018	0.113
	Time of measurement	1, 23	2.511	0.13	0.098	0.330
	Stimulation mode* Time of measurement	2, 46	1.613	0.21	0.066	0.324
Lapses	Stimulation mode	2, 46	1.359	0.27	0.056	0.278
	Time of measurement	1, 23	0.006	0.94	0.000	0.051
	Stimulation mode* Time of measurement	1.21, 27.78	0.805	0.40	0.034	0.179
Errors	Stimulation mode	1.45, 33.36	0.450	0.58	0.019	0.119
	Time of measurement	1, 23	3.728	0.07	0.139	0.456
	Stimulation mode* Time of measurement	1.52, 35.01	1.312	0.28	0.054	0.270

In terms of effect sizes (*d*_RM,pooled_), tDCS only had minimal effects on PVT measures of response speed (mean RT, SD RT) and small-to-medium but somewhat inconsistent effects on measures of response accuracy (lapses, errors; see Table 2). tRNS, on the other hand, had minimal-to-small effects on PVT measures of response speed in terms of faster and more consistent responding, but also had small effects on measures of response accuracy in terms of more erroneous responding. Sham stimulation had minimal-to-small effects on PVT measures of response speed (slower and more consistent responding) and only minor effects on measures of response accuracy (see Table 2).

### 3.3 Subjective sleepiness (KSS) and sleep quality

In terms of subjective level of sleepiness as assessed with the KSS, descriptive analyses pointed to lower scores (corresponding to heightened levels of wakefulness) after the tES sessions across all the stimulation modes (see Table 4). The conducted two-factorial RM-ANOVA corroborated this apparent effect, showing a significant main effect of Time of measurement (*p* = 0.01, partial-η^2^: 209; see also Table 5). Importantly, there was no significant interaction effect between Stimulation mode and Time of measurement and there was no main effect of Stimulation mode (Table 5). In terms of effect sizes (*d*_RM,pooled_), tRNS only had minimal, sham stimulation had small and tDCS had moderate favorable effects on subjective levels of sleepiness.

**Table 4:** Descriptive statistics of subjective sleepiness and sleep quality indicators before and after tES treatments (*M* ± *SD*; [Cohen’s *d*_RM, pooled_]; *n* = 29).

Outcome measure	tDCS		tRNS		Sham	
	Pre	Post	Pre	Post	Pre	Post
KSS	4.72 ± 1.69	3.90 ± 1.37	4.48 ± 1.64	4.28 ± 1.58	4.79 ± 1.72	4.21 ± 1.50
	[0.535]		[0.129]		[0.360]	
Sleep quality	–	3.00 ± 2.05	–	3.03 ± 1.88	–	3.03 ± 1.82

**Table 5:** Results of RM-ANOVAs with subjective sleepiness (KSS) and sleep quality (Likert scale) indicators.

	Factor	Test statistics				
		*df1, df2*	*F*	*p*	$\eta_{p}^{2}$	1-β
KSS	Stimulation mode	2, 56	0.387	0.68	0.014	0.109
	Time of measurement	1, 28	7.384	0.01	0.209	0.928
	Stimulation mode* Time of measurement	2, 56	1.190	0.31	0.041	0.250
Sleep quality	Stimulation mode	2, 56	0.008	0.99	0.001	0.051

The assessed sleep quality of the night sleep following each of the tES treatments showed almost identical values in descriptive terms (with a value of “3” corresponding to a rating of “mildly impaired”; see Table 4). The conducted RM-ANOVA showed no significant effect of stimulation mode on subjectively rated sleep quality (see Table 5).

### 3.4 Tolerability

There were no serious or major adverse reactions or events during or after tES treatment. Adverse reactions in the present study included itching and tingling sensations at the stimulation site in one single case (caused by both tDCS and tRNS) and, in one further case, a transient worsening of symptoms relating to restless legs syndrome (after tRNS). Apart from this, tDCS and tRNS did not impair subjective quality of nighttime sleep.

## 4 Discussion

In the present study, we examined the impact of single sessions of tDCS and tRNS on vigilance in patients suffering from narcolepsy and idiopathic hypersomnia. In contrast to the results presented by Frase et al. (28) and Galbiati et al. (30), there was no evidence for any significant short-term effect of anodal bifrontal tDCS on objective or subjective measures of sleepiness within the present study. We could also not demonstrate any acute effects of tRNS. Moreover, sleep quality during the night following the stimulation session was not affected neither by tDCS nor by tRNS.

One possible reason for the absence of therapeutic effects of tES on EDS in our study could be an insufficient statistical power. In our preliminary sample size planning, we assumed the occurrence of large effects (Cohen’s *d* ≥ 0.8) of active treatment conditions on both PVT responding and subjective levels of sleepiness. This assumption was grounded in the reports of very large effects (i.e., Cohen’s *d* > 1.5) of tDCS on subjective daytime sleepiness (30) and on objective vigilance (28). In their study, Frase et al. (28) reported a very large effect of tDCS on PVT reaction time. We estimated Cohen’s *d* > 2.8 from their data using an assumed correlation of *r* = 0.5 between the reported measurements (and the formula suggested by (81), p. 171). In terms of required sample size, we calculated that a sample size of *N* = 30 would be sufficient to detect large effects following the different stimulation modes (i.e., $\eta_{p}^{2}$ ≥ 0.14 for the interaction ‘Stimulation mode*Time of measurement’) in our two-factorial repeated measures design. As can be seen from the results of the conducted two-factorial RM-ANOVAS, the effect sizes for the comparison of stimulation modes on subsequent responding (i.e., the interactions ‘Stimulation mode*Time of measurement’) were all in the small-to-medium range (values of $\eta_{p}^{2}$ between 0.030 and 0.066, see Tables 3, 5). The calculated power indices (1-β) pointed to insufficient statistical power to detect statistically significant results given the small effect sizes of the present trial, especially for the interaction terms ‘Stimulation mode*Time of measurement’ (1-β ≤ 0.33; see Tables 3, 5). In addition, the effect sizes calculated for the single pre/post comparisons within stimulations modes (see Tables 2, 4) pointed toward mostly small effects of both tDCS and tRNS on subsequent sleepiness and vigilance. Moreover, there was no indication that any of the two tES stimulation modes yielded superior effects on subsequent responding. While tRNS had somewhat more consistent effects on PVT responding, leading to faster and more consistent reaction times (at the cost of more erroneous responses), tDCS had only minimal effects on PVT reaction times and inconsistent effects on response accuracy. Hence, the unexpectedly low effects of our employed single session treatments left us with insufficient statistical power to detect significant results of such (small) magnitude. Contrary to this lack of effects on objective indicators of sustained attention, tDCS had moderate positive effects on subjective levels of wakefulness, while sham stimulation had small and tRNS only had minimal effects (see Table 4). Nevertheless, there was a statistically significant main effect of Time of measurement, pointing toward significant increases in subjective levels of wakefulness independently from treatment condition. A possible explanation for these subjective increases relate to unspecific effects of stimulation (e.g., induced by expectation) that might be more pronounced at the subjective level. Another possibility for this increase in subjective levels of wakefulness relates to the occurrence of short-time sleep periods during the tES treatment sessions. Since we did not take measures to ensure wakefulness throughout the daily tES sessions, such sleep periods might have occurred and hence have led to subjective increases of wakefulness after the tES sessions. In fact, 13 patients (44.8%) were awake during the whole treatment time of both tDCS sessions. During the tRNS and sham stimulation, eight (27.6%) and six (20.7%) patients spent both sessions awake. While these differences between the three treatments were statistically significant (*Q* = 6.75, df = 2, *p* = 0.034), a *post hoc* correlational analysis of the association between sleep occurrence and the degree of differences in the outcome variables of interest (difference pre- to post treatment for all PVT measures and the KSS) showed mainly insignificant results (data not shown). The only significant association was between sleep during tRNS treatment and the difference in lapses in the PVT, in that there was a significantly reduced number of lapses after the stimulation session in those patients reporting sleep during the treatment period (*r* = 0.46, *p* = 0.025).

Another possible reason for the present null results of tDCS and tRNS relates to the dosing of tES stimulation. When compared to both Frase et al. (28) and Galbiati et al. (30), the present study employed the shortest tES treatment protocol with only single session treatments. It is possible that in the case study provided by Frase et al. (28), which was employed throughout a six-day period encompassing a total of three active treatment sessions with otherwise identical stimulation parameters (as compared to the present study), there were additive effects of tDCS across the treatment period. This interpretation is in line with the observed additional therapeutic effects of tDCS on subjective levels of sleepiness and duration of nighttime sleep throughout an extended treatment period in the case study presented by Frase et al. (28). However, since the vigilance of the patient was tested on a daily basis before and after each daily session (sham/active tDCS), the reported effects of tDCS must have occurred acutely after a single session. Likewise, the stimulation protocol used by Galbiati et al. (30) included repeated anodal tDCS over the left dorsolateral prefrontal cortex three times a week for a total duration of 4 weeks. As they were interested in additive effects of several sessions of tDCS treatment, the positive results presented by this group also add to the notion of possible additive effects of tDCS on sleepiness in hypersomniac patients. We chose for the investigation of “bottom-line” effects of single tES sessions on objective indicators of sustained attention against the background of robust evidence for such acute effects in healthy subjects (31, 62, 63, 65). Contrary to healthy subjects, the majority of patients enrolled in the present study (75.9%) received pharmacological treatment for their EDS symptoms and had a rather long history of formal diagnosis (~ 8.5 years before study entry). Therefore, the reduced reactivity of our sample to the employed tES protocol might also be due to the medicated state and a potentially resultant lessened reactivity of the patients’ brains to tES. Moreover, the postulated hypofrontality in EDS disorders (21) could be related to reduced reactivity of the frontal cortex to tES effects in the population of interest. This understudied topic clearly deserves further research in order to arrive at a better understanding for the therapeutic potential of tES in EDS disorders.

Lastly, another reason for the present null results refers to the disease entities studied and their underlying (partly unclear) neurobiological underpinnings. tES is a neuromodulatory technique with a limited focality, not only affecting specific target regions of the human brain, but rather widespread neural circuits (32). The stimulation protocol used by Frase et al. (28) was used for treating a patient with isolated organic hypersomnia following reanimation. In this context, it is important to note that the brain regions involved in organic forms of hypersomnia resulting from traumatic brain injury (82, 83) differ significantly from other forms of hypersomnia (9, 13, 84). Organic hypersomnia is often caused by thalamic or brainstem strokes (82, 83). With differences in the pathophysiology of the different types of hypersomnia, the results from the case report of Frase et al. (28) might not be transferrable to our patient cohort, which comprised patients with narcolepsy (NT 1, NT 2) and IH. Since we intermixed different subgroups of patients in the presented statistical analysis, we cannot rule out the possibility of more pronounced tES effects at the level of specific diagnostic subgroups. We abstained from a subgroup-specific analysis due to limited statistical power. Based on a recent review of neuroimaging studies pointing at hypofrontality across diagnostic subgroups of EDS disorders (21), together with the rationale of tES-induced effects on a “top-down” network for sleep regulation (60), we chose this syndrome- and not etiology based approach as a first step. Future studies might focus on tES effects on specific diagnostic subgroups of patients with EDS in order to provide more insight to this important research question.

Nonetheless, the validity of the present null results should also be considered. While differing from previous studies, the present null findings might also be due to the application of sound study procedures. Previous publications could be confounded with methodological limitations, Galbiati et al. (30) used an uncontrolled study design that is susceptible for potential placebo effects. The case report of Frase et al. (28) included only a single patient and therefore only has very limited generalizability. Moreover, the statistical design of their study seems somewhat flawed (i.e., they conducted a RM-ANOVA and *post hoc* testing based on data of a single case). Our study procedures tried to control for potential placebo effects by employing a double-blind, pseudo-randomized crossover design with a sham control condition. While 44.8% of our sample was able to correctly identify the sham condition, the tRNS condition was mistakenly regarded as sham treatment by 37.9% of patients. Only 17.8% mistakenly regarded the tDCS session as the sham treatment, pointing to partial success of our blinding procedure. Therefore, future studies of tDCS effects in hypersomniac patients should take care to control for these important effects in order to provide sound empirical evidence of therapeutic effects. In case of future studies, the present results clearly point toward the need of repeated sessions of the different treatment modalities, hence enabling the occurrence of additive effects of tES. In line with previous studies (28, 30), we observed a favorable side-effects profile of tDCS and tRNS within our patient sample. Future studies might attempt to implement control procedures in order to avoid episodes of short-time sleep during the tES treatment sessions.

Further research on non-drug approaches in patients with EDS such as tES is required to investigate whether – and to what extent – a modification of the stimulation parameters used in the present study could improve sleepiness in patients with hypersomnia. As cumulated therapy effects of tDCS and tRNS are suggested in numerous previous studies on healthy adults (38, 46) and patients with EDS (28, 30), further tES study protocols for reducing sleepiness should be conducted, employing repeated stimulation sessions and larger patient collectives. Furthermore, the evaluation of the stimulation protocol used in the trial of Galbiati et al. (30) by means of a double-blind sham-controlled randomized study design could be helpful to generate new knowledge on tES in the treatment of EDS.

## Data availability statement

The raw data supporting the conclusions of this article will be made available by the authors, without undue reservation.

## Ethics statement

The studies involving humans were approved by Ethics Committee of the University of Regensburg (Universität Regensburg, 93040 Regensburg, ethikkommission@ur.de, reference: 15-101-0295). The studies were conducted in accordance with the local legislation and institutional requirements. The participants provided their written informed consent to participate in this study.

## Author contributions

MH: Conceptualization, Data curation, Investigation, Methodology, Writing – original draft, Formal analysis. BL: Conceptualization, Resources, Writing – review & editing. TW: Conceptualization, Supervision, Writing – review & editing. PG: Conceptualization, Project administration, Resources, Supervision, Writing – review & editing. MS: Conceptualization, Writing – review & editing, Project administration, Resources, Supervision. AR: Conceptualization, Data curation, Investigation, Methodology, Writing – original draft, Writing – review & editing.
